# 

**Published:** 1890-03

**Authors:** 


INTERNATIONAL DENTAL JOURNAL

FOR 1890.

Its fearless, independent course in defence of professional interests should make it indispensable to every dentist.

ALL DEALERS ARE OUR AUTHORIZED AGENTS. TERMS, $2.50 PER ANNUM, IN ADVANCE.

